# An optical study of drug resistance detection in endometrial cancer cells by dynamic and quantitative phase imaging

**DOI:** 10.1002/jbio.201800443

**Published:** 2019-04-02

**Authors:** Tian Yao, Runyu Cao, Wen Xiao, Feng Pan, Xiaoping Li

**Affiliations:** ^1^ Department of Obstetrics and Gynecology Peking University People's Hospital Beijing China; ^2^ Key Laboratory of Precision Opto‐Mechatronics Technology of Ministry of Education, School of Instrumentation Science & Optoelectronics Engineering Beihang University Beijing China

**Keywords:** digital holographic microscopy, drug resistance, endometrial cancer, quantitative phase imaging

## Abstract

Platinum chemosensitivity detection plays a vital role during endometrial cancer treatment because chemotherapy responses have profound influences on patient's prognosis. Although several methods can be used to detect drug resistance characteristics, studies on detecting drug sensitivity based on dynamic and quantitative phase imaging of cancer cells are rare. In this study, digital holographic microscopy was applied to distinguish drug‐resistant and nondrug‐resistant endometrial cancer cells. Based on the reconstructed phase images, temporal evolutions of cell height (CH), cell projected area (CPA) and cell volume were quantitatively measured. The results show that change rates of CH and CPA were significantly different between drug‐resistant and nondrug‐resistant endometrial cancer cells. Furthermore, the results demonstrate that morphological characteristics have the potential to be utilized to distinguish the drug sensitivity of endometrial cancer cells, and it may provide new perspectives to establish optical methods to detect drug sensitivity and guide chemotherapy in endometrial cancer.

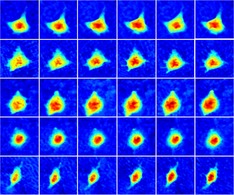

## Graphical abstract

Drug resistance detection is crucial for cancer treatment. This research aims at revealing relations between drug resistance of cancer cell and cellular morphology. The morphological changing process of drug‐resistant and nondrug‐resistant endometrial cancer cell are measured by digital holographic microscopy and results show that cells with different degree of drug resistance have distinct morphological changes exposing to cisplatin. The research may provide new perspective to guide drug resistance detection.

## 1 INTRODUCTION

Endometrial cancer is one of the most commonly diagnosed gynecological malignancies 1, 2. The main treatment of endometrial cancer is adjuvant platinum‐based chemotherapy and radiotherapy after surgery. The recurrence rate of advanced endometrial cancer is as high as 50%, and the 5‐year survival rate of patients with recurrent endometrial cancer is only 20% to 40% 3, 4, 5. Emerging evidence indicates that endometrial cancer recurrence is closely related to chemotherapy resistance 6, 7. Thus, the assessment of cancer chemosensitivity plays an important role in cancer treatment process. At present, the assessment of chemotherapy sensitivity in patients with endometrial cancer mainly depends on the clinical experience of the tumor physicians based on physical examination, gynecological examination, medical imaging techniques, including magnetic resonance imaging, computed tomography 8, or the identification of tumor biomarkers, such as cancer antigen 125 9, 10. However, the use of these methods to estimate drug sensitivity has been disappointing. Therefore, it is of great importance to accurately determine the sensitivity of patients to chemotherapeutic drugs and implement individualized chemotherapy.

The common prediction model of tumor drug resistance has been used in recent years to establish a biological model based on tumor tissues such as transplanting tumor tissue or purified tumor cells into nude mice to observe the chemotherapeutic responses. However, due to the low success rate, high costs and time required to establish the biological model, the clinical application of this method is limited 11, 12. Cell line models are another essential preclinical tumor model 12, 13, 14 that can only be used to examine drug sensitivity by evaluating of the metabolic activity of cells through methyl tetrazolium (MTT) assays, as a reflection of cell viability. RNA sequencing is another commonly used method to predict drug sensitivity by detecting multiple drug resistance genes. However, MTT can only be used to evaluate purified tumor cells, which require a long time to establish. Thus, because of its low sensitivity, the ability for RNA sequencing to predict drug sensitivity after surgery is limited.

In cancer cells, exposure to cisplatin may cause changes in cell morphology in a short period of time 15, 16, which may be related to toxicity caused by oxidative stress, DNA damage and/or cell death via apoptosis or necrosis 17, 18. Much work has been performed to quantify the effect of drugs on cancer cells such as fibroblasts 19, prostate cancer cells 20 and ovarian cancer cells 21.

These researches have demonstrated the possibility of optical studies to be used in evaluating tumor cell drug sensitivity characteristics. Thus, if the optical methods can help doctors to distinguish drug‐resistant and nondrug‐resistant cells, the drug sensitivity characteristics of different patients may be diagnosed in a more convenient way and within a shorter time.

Digital holographic microscopy (DHM) has been widely used in recent years to monitor the dynamic morphological changes of living cells 22. As an innovative quantitative phase imaging approach, DHM is capable of recording the optical path distributions of samples with just a single exposure, making it an ideal method to retrieve dynamic processes, especially for living cells. As the illumination, intensity in the DHM setup is relatively low and the light source, which is generally a coherent laser, has no phototoxicity, living cells can be and tracked for several hours in real time. Furthermore, no labeling or staining is necessary for DHM measurement. Due to advances in living cell monitoring, DHM has been widely used in biomedical research 23, 24, 25, 26, 27, especially for measuring on morphological changes in living cells 28, 29, 30, 31, 32, 33.

In this study, we aimed to establish an optical method at the single‐cell level to detect the drug resistance of endometrial cancer cells. DHM was applied to dynamically and quantitatively record and reconstruct the phase images of the cells and to retrieve the temporal evolutions of cell morphological parameters, which were used to distinguish between the drug‐resistant and nondrug‐resistant endometrial cancer cells. Cell morphological parameters, namely, cell height (CH), cell projected area (CPA) and cell volume (CV), were calculated from the reconstructed phase images. After cisplatin treatment, the change rates of morphological parameters between Ishikawa (ISK) and ISK/CisR (ISK‐CisR) cells were significantly different and the results were consistent with the biomedical experiments.

## 2 METHODS

### 2.1 Cell culture and treatments

The human endometrial cancer cell line ISK is a well‐differentiated adenocarcinoma, estrogen receptor α (ERα)(+), ERβ(+) and progesterone receptor cell line derived from the American Type Culture Collection that was preserved in the Obstetrics and Gynecology Laboratory of Peking University People's Hospital. ISK‐CisR cells were successfully induced by exposing ISK cells to cisplatin for 10 months to obtain drug resistance characteristics 34. Both ISK and ISK‐CisR cells were cultured and grown in Dulbecco's Modified Eagle's Medium/Ham's F‐12 50/50 Mix (CORNING, Corning, New York) supplemented with l‐glutamine, 15 mM HEPES (2‐[4‐(2‐Hydroxyethyl)‐1‐piperazinyl] ethanesulfonic acid) and 10% fetal bovine serum (Gibco 10099‐141, Australia) at 37°C and CO_2_. Cells were seeded onto a 35 mm glass WillCo dish at a density of 5 × 10^3^ cells per plate. After 24 hours, cisplatin (Sigma‐Aldrich, Steinheim, Germany) was added to the medium at a final concentration of 5 μg/mL (low concentration [LC] medium) or 100 μg/mL (high concentration [HC] medium). The negative controls contained dimethyl sulfoxide (DMSO) (control medium).

### 2.2 Biological characteristics of ISK and ISK‐CisR cells

#### 2.2.1 Determining the resistance index of endometrial cancer cell lines ISK‐CisR compared to ISK cells

The cultured ISK and ISK‐CisR cells were collected, the cell density was adjusted to 3 × 10^5^ cells/mL, and cells were inoculated into 96‐well plate. Each cell type was divided into 10 groups, and each group had six wells. The zero well (ie, the blank group) was included (with medium only) with a volume of 100 μL/well. The cells were placed in a humidified incubator at 37°C with 5% CO_2_ for culturing, and when the cells were attached, the medium was replaced with 100 μL culture medium containing different concentrations of cisplatin (0, 5, 10, 20, 50, 100, 200, 400, 800 and 1600 μg/mL). The media of the blank and control groups were replaced with fresh culture medium without cisplatin, and after culturing for 48 hours, the number of live cells was determined by the Cell Counting Kit‐8 (CCK‐8; Dojindo, Kumamoto, Japan). CCK‐8 allows very convenient assays by utilizing highly water‐soluble tetrazolium salt. Water‐soluble tetrazolium‐8 (WST‐8) produces a water‐soluble formazan dye upon reduction in the presence of an electron mediator, and the amount of formazan produced is proportional to the number of living cells.

#### 2.2.2 Determining the rate of apoptosis in ISK and ISK‐CisR endometrial cancer cell lines

To assess the apoptotic sensitivity induced by cisplatin, ISK and ISK‐CisR cells were treated with control medium, LC and HC for 2 hours. Next, cancer cells were collected from wells using trypsin digestion, washed in phosphate‐buffered saline (PBS) and then suspended in 500 μL of 1× binding buffer (10 mM HEPES, 140 mM NaCl, 2.5 mM CaCl_2_, pH 7.4) with 10 μL of annexin conjugate and PI. After incubation for 15 minutes, at least 10^4^ cells were analyzed using a three laser/six color Becton‐Dickinson LSR II.

#### 2.2.3 Determining F‐actin distribution in ISK and ISK‐CisR endometrial cancer cell lines

The expression level of F‐actin in ISK and ISK‐CisR cells was determined by immunofluorescence staining. After the addition of control medium, LC and HC, the cells were briefly washed with PBS three times and fixed with 4% paraformaldehyde for 20 minutes. Phalloidin‐rhodamine (Invitrogen, Carlsbad, California) was used to label F‐actin (1:500 solution for 1 hour), and 4′,6‐diamidino‐2‐phenylindole (1:1000) (Invitrogen) was used to label nuclei (1:1000 solution for 5 minutes). For the observation, a laser scanning confocal microscope was used (Leica Microsystems, Wetzlar, Germany).

### 2.3 DHM setup and data processing

#### 2.3.1 DHM setup

**Figure 1:**
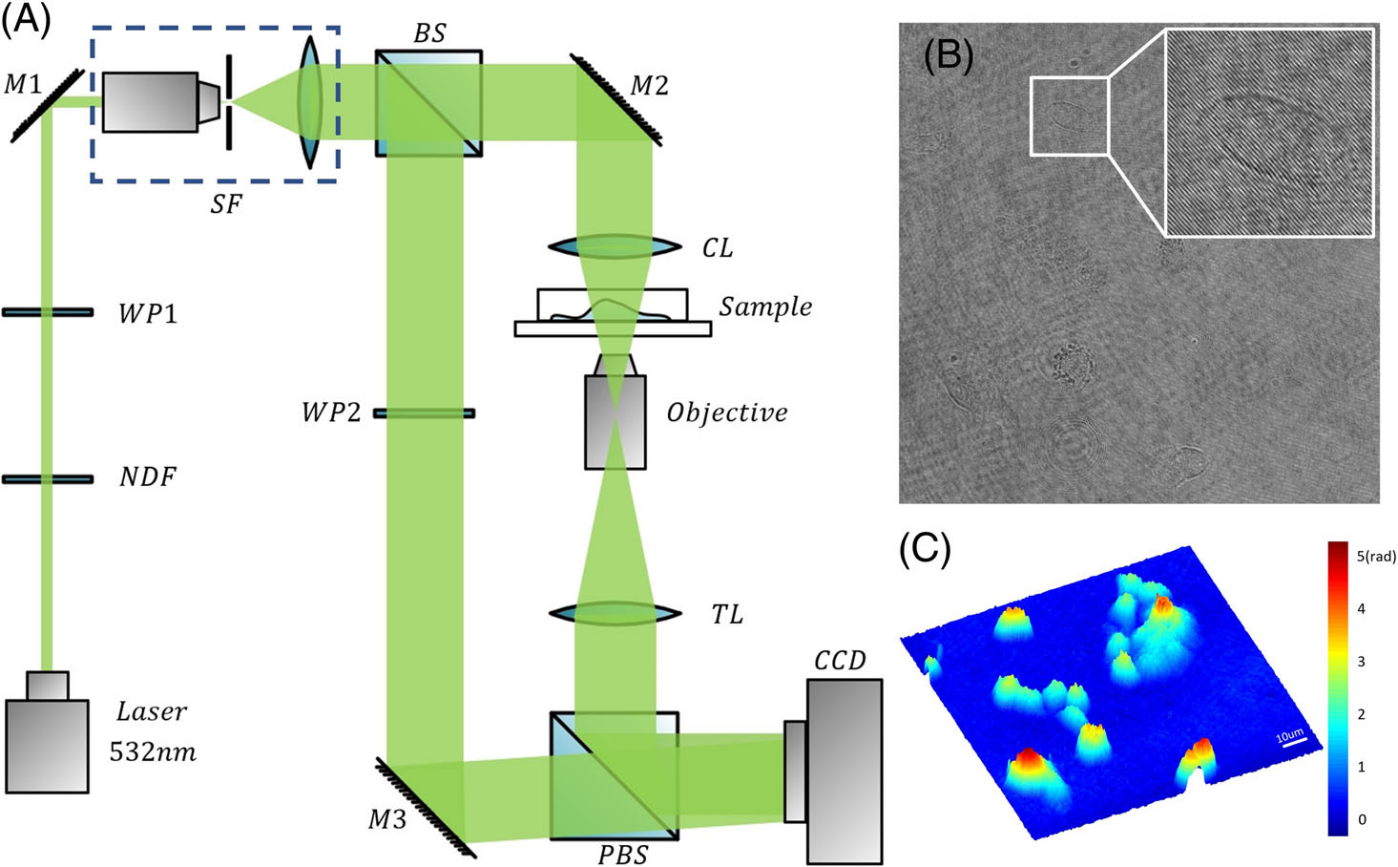
(A) Schematic of the DHM system; (B) a single hologram with a dimension of 512 × 512 pixels; and (C) a reconstructed pseudo‐3D phase map from (B); the white line indicates 10 μm and the color bar is shown on the right

In this research, holograms of ISK cells were recorded by a typical off‐axis DHM setup, which has been fully studied and depicted in References 35, 36, 37. The schematic of the DHM configuration is shown in Figure 1A. The coherent light source was generated by a 532 nm laser (Cobolt, Solna, Sweden) with an output power of 100 mW. To avoid hurting the living cancer cells, a neutral density filter was placed after the laser to attenuate the light power. After propagating through the spatial filter, the expanded laser beam was split into an object beam and a reference beam by a polarizing beamsplitter. The object beam was converged by a condenser lens and then transmitted through the samples. To obtain optimal imaging resolution, the wave front was magnified by an objective (Olympus UPLFLN, 20×, NA = 0.50) combined with a tube lens. The amplified object beam interfered with the reference beam at the camera plane and the holograms were recorded by a charge‐coupled device (CCD) camera (1024 × 1024 pixels, 5.86 μm, PointGrey, Canada). A single hologram and the reconstructed pseudo‐three‐dimensional (3D) phase maps are shown in Figure 1B,C. From Figure 1C, it can be seen that CH in rad ranges from about 2 to 4 rad depending on cell status.

#### 2.3.2 Hologram recording and phase reconstruction

To fully retrieve the different morphological temporal evolutions of ISK cells with and without cisplatin, the hologram was recorded every 5 seconds and continuously recorded 150 minutes after drug treatment (a total of 1800 frames).

After hologram recording, the angular spectrum method was applied to reconstruct the phase images, and the Zernike polynomial fitting method was used to compensate the tilt aberrations caused by the off‐axis geometry and the high‐order curving phase aberrations induced by the objective. An offset procedure was applied to the reconstructed phase images to make the average background phase value near the zero line.

To validate the DHM setup, a temporal stability test was conducted before the experiments. A set of blank phases was recorded for 10 minutes with the same frame rate, a region with 3 × 3 pixels was randomly chosen and the temporal SD of the phase value in the region was 0.43° to 0.61°. Under a laser wavelength of 532 nm and when the refractive index (RI) difference between the ISK cells and culture was 0.05, the temporal stability of the DHM setup was in the precision of 100 nm, which was far less than the expected basal CH change range (micrometer scale) 38, and the change in CH would be more active when resisting drugs.

### 2.4 Calculation and analysis of cellular morphological parameters

To assess the morphological changing process between ISK and ISK‐CisR cells, three cell parameters were considered: CH, CPA and CV. All parameters are fully defined and described in the following section.

#### 2.4.1 Calculation of morphological parameters

To quantitatively measure the change in cell morphology, three parameters were taken into consideration and calculated from the reconstructed phase distributions.

In DHM measurements, phase shift data were calculated from Eq. (1):

(1) $$\Delta \varphi \left(x,y\right)=\frac{2\pi }{\lambda }\left[n_{c}\left(x,y\right)-n_{m}\right]h\left(x,y\right),$$

where λ is the laser wavelength, *n*_*c*_(*x*, *y*) is the cellular RI, *n*_*m*_ is the RI of the surrounding solution and *h*(*x*, *y*) is CH. From Eq. (1), it can be seen that the cell phase value at (*x*, *y*) is coinfluenced by the cellular RI and CH. Since the change in the cellular RI is too slight to affect the change trend of CH, the phase value is considered to have the same change trend as CH. According to this principle, CH is directly represented by the phase value.

CPA and CP reflect both the transverse and 3D morphology of the living cell. Before calculating these two parameters, an automatic cell edge detection procedure is conducted to determine the cell profile. Once single cells are determined, CPA and CV can be calculated by Eqs. 2, 3, as follows:

(2) $$S_{\text{cell}}=A\times \mathrm{Nu}m_{\text{cell}.}$$

(3) $$V_{\text{cell}}=S_{\text{cell}}\times {\bar{\varphi }}_{\text{cell}.}$$

In these two equations, Num_cell_ denotes the number of pixels within the cell, ${\bar{\phi }}_{\text{cell}}$ denotes the average phase value within the cell and *A* denotes the real area of one pixel of the CCD camera. Thus, the projected area of a cell, *S*_cell_, is defined as the sum of the real pixel areas within the cell and CV, *V*_cell_, is calculated by multiplying the projected area and the mean phase value within the cell.

#### 2.4.2 Change rate calculation

To quantitatively compare the changing process, the change rates of CH, CPA and CV are calculated according to their temporal evolutions, which are defined as Eq. (4):

(4) $${\mathrm{CR}}_{\mathrm{CH}}=\frac{{\bar{\mathrm{CH}}}_{l5}-{\bar{\mathrm{CH}}}_{f5}}{{\bar{\mathrm{CH}}}_{f5}}\times 100\%.$$

In Eq. (4), CR_CH_ represents the change rate of CH, ${\bar{\mathrm{CH}}}_{l5}$ represents the mean CH in the last 5 minutes and ${\bar{\mathrm{CH}}}_{f5}$ represents the mean CH in the first 5 minutes. It can be seen from Eq. (4) that a change rate in this research is defined as the proportion of variations in values of the first and last 5 minutes to the average value in the first 5 minutes. Change rates of all the three parameters are calculated in each group with the statistical error.

#### 2.4.3 Statistical analysis

All biological experiments were performed in triplicate and repeated three times. During time‐lapse imaging, for any set, at least 10 cells were imaged for each condition. GraphPad 7.0 was used for all statistical analyses. For comparisons between populations of cells, Student's *t* test was used to compare the differences between two groups, and *P* < 0.05 was considered statistically significant.

## 3 RESULTS

### 3.1 The resistance index of ISK‐CisR cells compared to ISK cells

**Figure 2:**
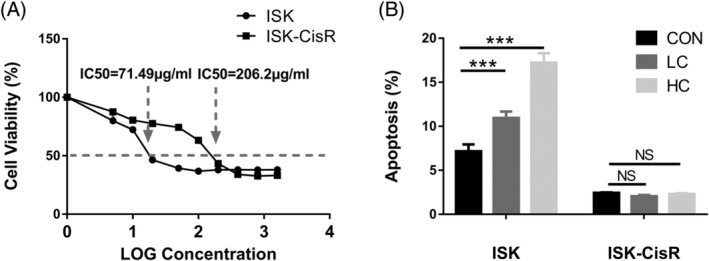
Biological characters of ISK and ISK‐CisR cells: (A) IC50 values of ISK and ISK‐CisR cells and (B) apoptotic rate of ISK and ISK‐CisR cells

The endometrial cancer cisplatin‐resistant cell line ISK‐CisR was successfully induced by exposing ISK cells to cisplatin for 10 months. CCK8 tests were used to calculate the drug resistance index. As shown in Figure 2A, the IC50 value was the concentration of cisplatin that induced 50% cell death. The IC50 value of ISK cells was 71.49 μg/mL, while that of ISK‐CisR cells was 206.2 μg/mL. Thus, the resistance index (IC50 value of ISK‐CisR cells/IC50 value of ISK cells) of ISK‐CisR cells was 2.89, suggesting that ISK‐CisR cells acquired cisplatin resistance.

### 3.2 The apoptotic rate of ISK and ISK‐CisR cells following treatment with different concentrations of cisplatin

Based on the published study in Reference 19, cytotoxicity‐induced cell morphological changes might be related to cellular apoptosis. To further confirm the apoptotic rate of ISK and ISK‐CisR cells induced by different concentrations of cisplatin, the apoptotic cells were counted after 2 hours of cisplatin treatment. As shown in Figure 2B, the apoptotic rate was increased in ISK cells following LC and HC treatment compared to the control medium group (10.94% vs 7.15%, respectively; *P* < 0.05, and 17.2% vs 7.15%, respectively; *P* < 0.05) but was not altered in ISK‐CisR cells (2.1% vs 2.44%; *P* > 0.05, and 2.36% vs 2.44%; *P* > 0.05).

### 3.3 Determining F‐actin distribution in ISK and ISK‐CisR endometrial cancer cell lines

**Figure 3:**
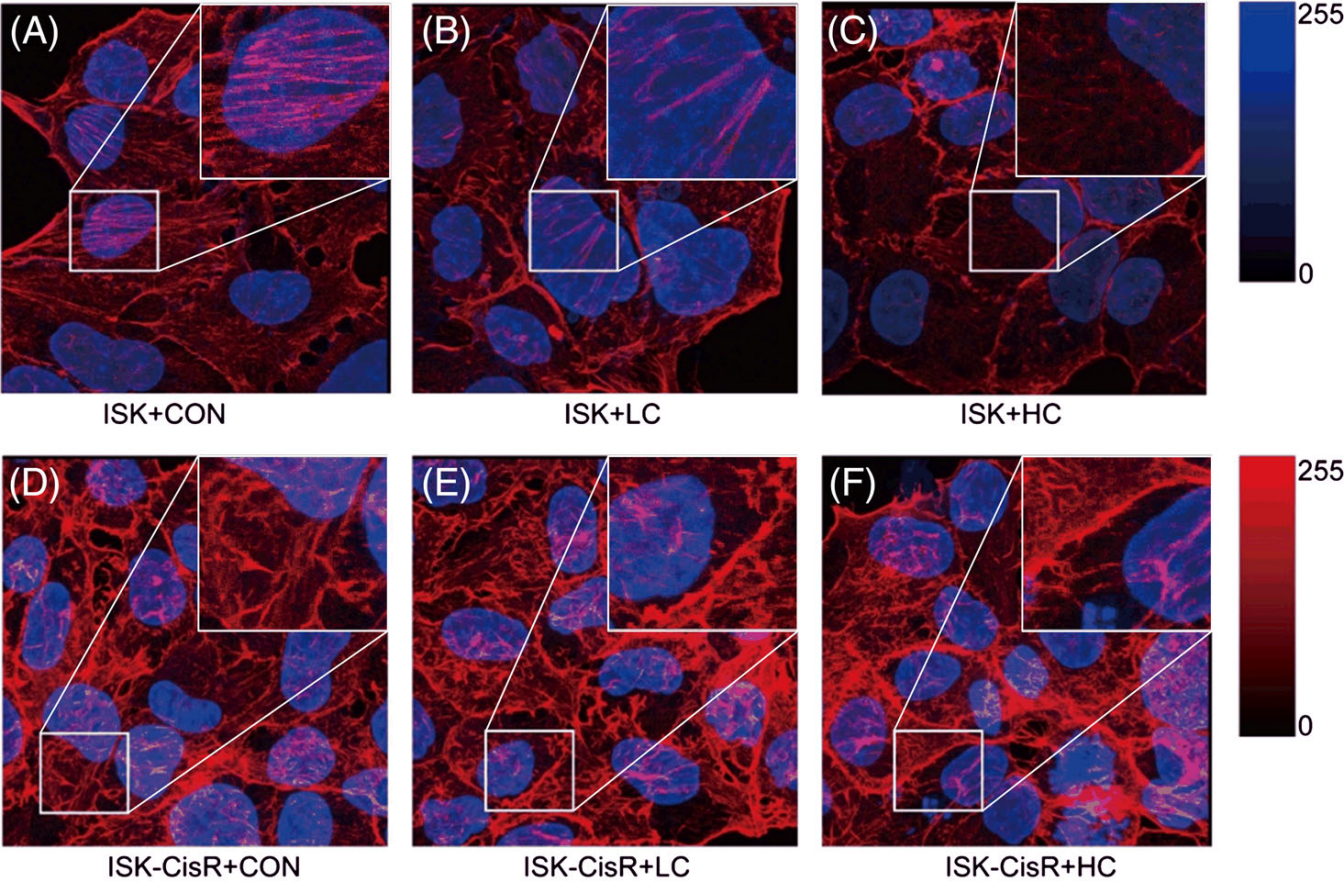
F‐actin distribution of ISK and ISK‐CisR cells following treatment with different concentrations of cisplatin: (A) ISK + CON; (B) ISK + LC; (C) ISK + HC; (D) ISK‐CisR + CON; (E) ISK‐CisR + LC; and (F) ISK‐CisR + HC

To determine whether actin stress fibers were a distinguishing feature of platinum‐resistant cells, we examined F‐actin cytoskeletal organization in ISK and ISK‐CisR 39. Since cisplatin can induce F‐actin damage, we investigated the F‐actin distributions of ISK and ISK‐CisR cells following treatment with different concentrations of cisplatin. As shown in Figure 3, all pictures were captured in the same condition and the details of F‐actin fibers were marked in white boxes. The F‐actin fibers of ISK cells (a) were long and regular, while the fibers of ISK‐CisR cells (d) were short and reorganized. After cisplatin treatment with LC medium, the F‐actin fibers of ISK cells (b) were thinner while in ISK‐CisR cells (e), F‐actin fibers were unchanged. After cisplatin treatment with HC medium, the F‐actin fibers of ISK cells were fractured and long actin fibers disappeared in ISK cells (c), while the F‐actin fibers of ISK‐CisR cells were stable (f).

### 3.4 Changed trends and rates of the morphological parameters

**Figure 4:**
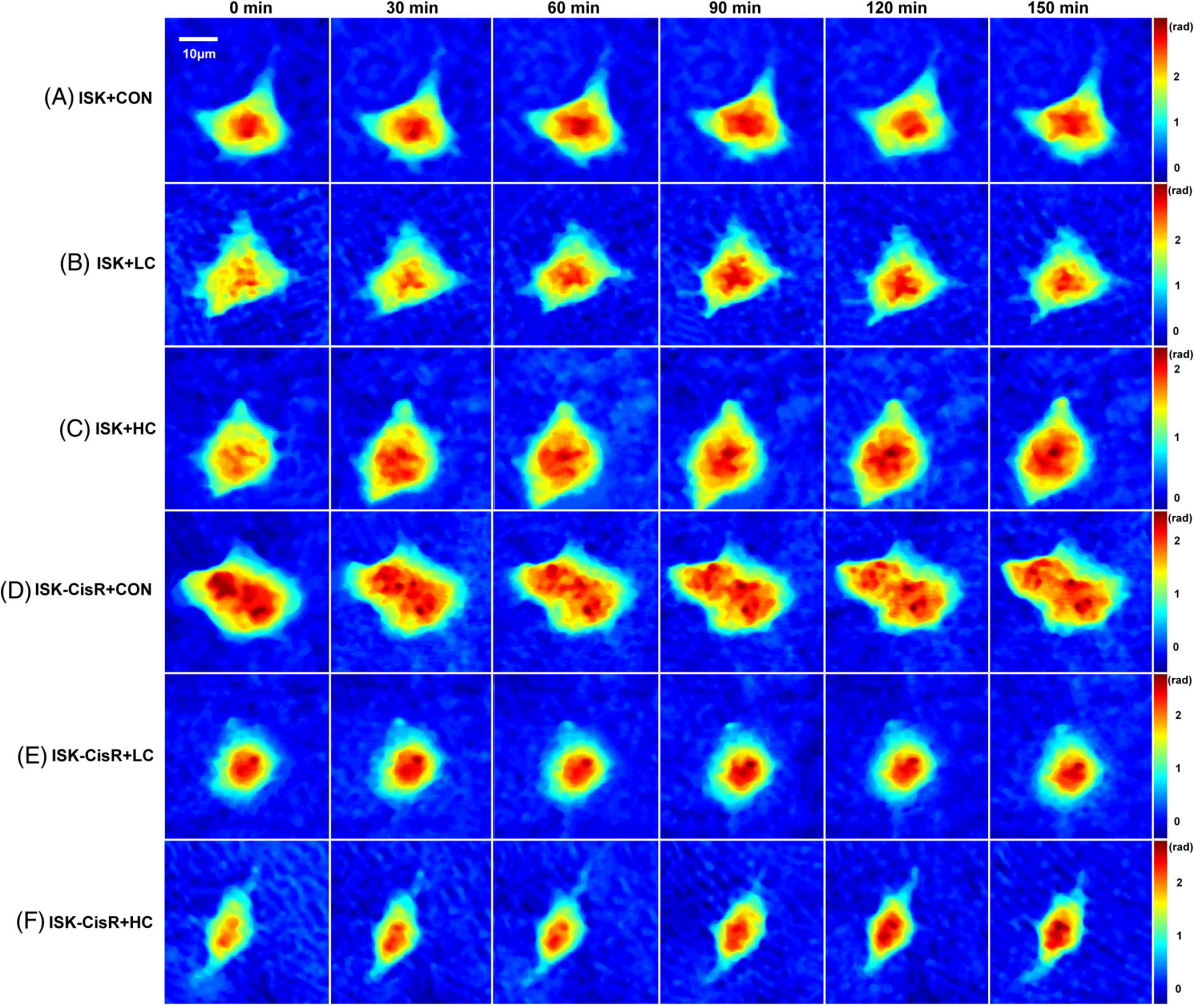
Consecutive holographic phase images over a 150‐minute period for (A) ISK cells in control group, (B) ISK cells treated with LC media, (C) ISK cells treated with HC media, (D) ISK‐CisR cells in control group, (E) ISK‐CisR cells treated with LC media, and (F) ISK‐CisR cells treated with HC media

ISK and ISK‐CisR cells were individually treated with LC or HC medium, and ISK cells were treated with control medium as the control. All groups were cultured under the same environmental conditions and in each group 150 minutes of recording was conducted. As shown in Figure 4, a set of holographic phase images over a 150‐minute period for control group, ISK and ISK‐CisR with LC and HC treatment were displayed, respectively. Phase maps at 0, 30, 60, 90, 120, and 150 minutes of each group were displayed with a unified color map. The white line in the first block indicated the scale of the phase maps, and color bars of each series of maps were located at the right. According to Figure 4, no obvious change in phase value and cellular shape was found in control group, ISK‐CisR with LC group, while slight changes were found in ISK with LC group and ISK‐CisR with HC group. As we expected, the most significant changes in CH and cellular morphology were found in ISK with HC group.

**Figure 5:**
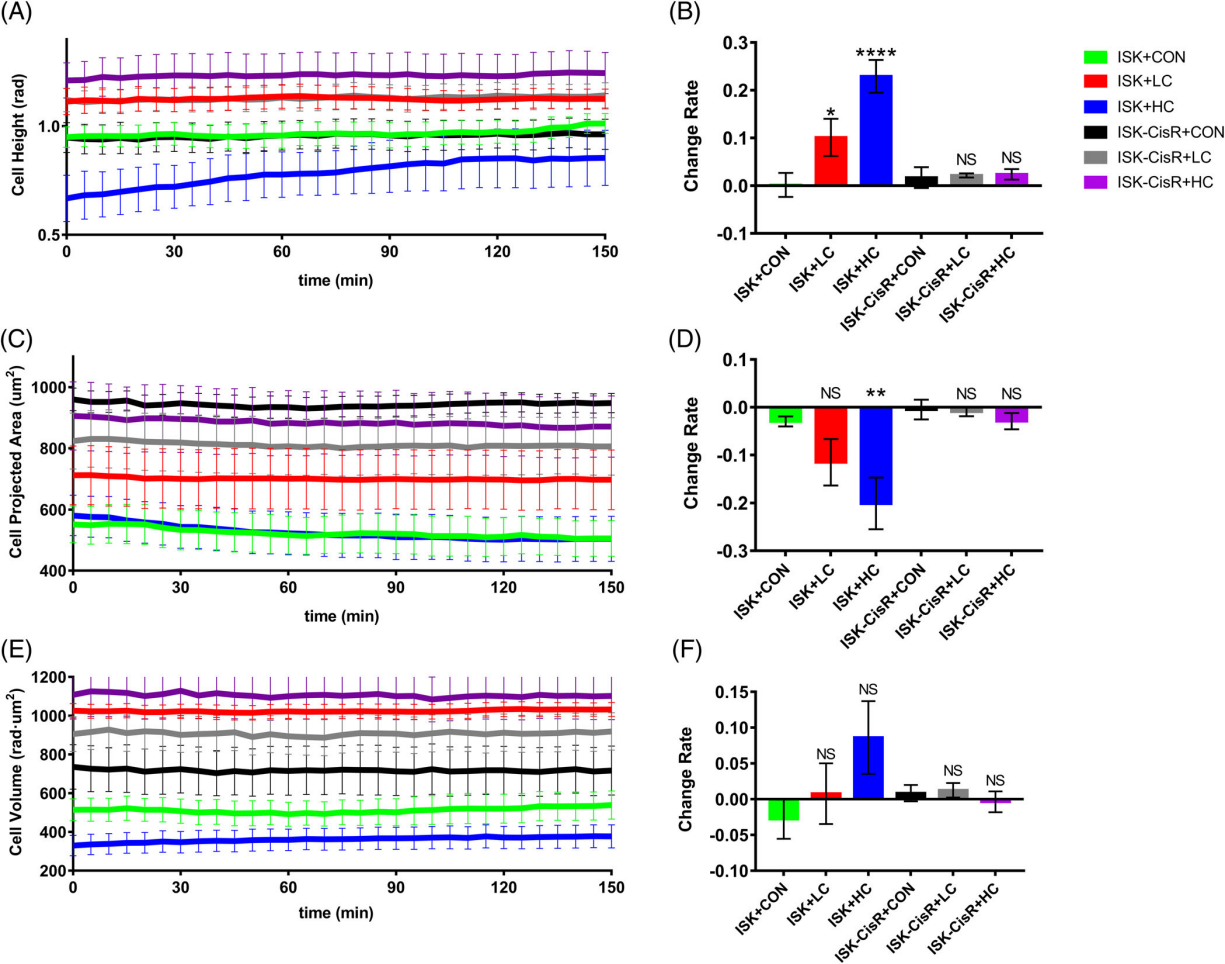
Temporal evolution of (A) CH, (C) CPA and (E) CV, (B‐F) change rate of the three morphological parameters. Green lines and bars represent the ISK control group, red represents the ISK group with LC medium, blue represents the ISK group with HC medium, black represents the ISK‐CisR control group, gray represents the ISK‐CisR group with LC medium and purple represents the ISK‐CisR group with HC medium

The changes in CH, CPA and CV were quantitatively examined before and after exposure to cisplatin. We hypothesized that these cell lines might develop resistance to cisplatin after 10 months of exposure and that the behavior would be associated with additional morphological changes. In particular, a distinct change in CH, CPA and CV of ISK and ISK‐CisR cells following treatment with different concentrations of cisplatin was expected. The temporal evolutions of CH, CPA and CV and the change rates of these three parameters are presented in Figure 5. As shown in Figures 4B and 5A, the CH of ISK cells with LC and HC treatment increased significantly (10.1% vs 0.16%, *P* = 0.0460 and 22.9% vs 0.16%, *P* < 0.0001, respectively) compared to the control group, while for ISK‐CisR cells, the height remained nearly unchanged following LC and HC treatment (2.2% vs 0.16%, *P* = 0.4417 and 2.4% vs 0.16%, *P* = 0.4293, respectively). As shown in Figures 4D and 5C, the CPA of ISK cells with HC treatment decreased significantly compared to the control group (20.1% vs 2.96%, respectively, *P* = 0.0059), while these changes were not observed in ISK cells following LC treatment (11.5% vs 2.96%, respectively, *P* > 0.1039) or in ISK‐CisR cells with LC (0.9% vs 2.96%, respectively *P* = 0.1687) and HC (2.9% vs 2.96%, respectively, *P* = 0.9782) treatment. As shown in Figures 4F and 5E, the CV of ISK and ISK‐CisR cells did not change significantly regardless of LC (0.8% and 8.6% vs 2.79%, *P* > 0.1) or HC (1.2% and 0.3% vs 2.79%, *P* > 0.05) treatment.

## 4 DISCUSSION

Cisplatin is one of the most commonly used chemotherapies in gynecologic oncology. Unfortunately, many cancers initially respond well to platinum treatment but often develop resistance when tumors recur 40, 41, 42. Until now, studies have shown that increased DNA repair, altered cellular accumulation, and increased drug inactivation are the main reasons for cells to acquire drug resistance 17. Although several methods are used to detect the drug resistance characteristics of cancer cells, studies on the chemosensitivity of tumor cells based on dynamic and quantitative phase imaging are rare.

In this research, morphological changes caused by HC and LC treatment were used to differentiate between ISK and ISK‐CisR cells. Several morphological parameters of endometrial cancer cells were quantified, and significant changes in the parameters were observed. Aiming at differentiating ISK and ISK‐CisR cells through morphological characteristics, CH, CPA and CV are chosen and calculated based on phase maps, in that the three parameters are capable of directly describing the morphological characteristics of cell shapes. Besides, the three parameters can be directly calculated from the phase map, inducing no extra calculation or simulation errors. Specifically, following cisplatin treatment, the CH of nondrug‐resistant cells increased gradually and the CPA decreased gradually, while the CV did not change significantly. The CH change rate could be used to distinguish between drug‐resistant and nondrug‐resistant cells following LC and HC treatment, while the CPA change rate could only be used to differentiate between the two cell types following HC treatment. However, the CV did not change significantly following LC and HC treatment.

Cisplatin exposure was responsible for inducing the cell morphological changes and apoptosis. The apoptotic cells had typical morphological changes, such as cytoplasmic shrinkage, the leakage of intracellular liquids and successive membrane rupture during apoptosis, leading to an increase in CH and a decrease in CPA. The apoptosis activity of the two cell types changed before and after LC and HC treatment (Figure 1B). A previous study reported that changes in CH, CPA and CV were closely related to cellular apoptosis activity 19. One possible reason for the nearly unvarying CV change rates may be that the cisplatin treatment time was too short to induce a change in volume, but this explanation requires further investigation.

It has also been reported that cisplatin resistance in cancer cells is highly associated with cytoskeletal reconstruction. Cytoskeletal reconstruction leads to changes in cell morphology, producing biological characteristic changes in cancer cells with drug resistance. In our study, the structural changes of F‐actin were observed by immunofluorescence staining (Figure 3). In ISK cells, F‐actin was damaged after LC and HC treatment, while F‐actin expression did not change significantly after LC and HC treatment in the ISK‐CisR group. Sharma et al.^21^ also reported that cisplatin drug resistance was related to differences in the actin cytoskeleton. Cisplatin‐resistant ovarian cancer cell lines OVCAR5‐CisR and SKOV3‐CisR6 have more robust actin cytoskeleton and stress fibers than their isogenic cisplatin‐sensitive cell lines OVCAR5 and SKOV3.6. The cytoskeleton is the basic structure of cell morphology; thus, when stimulated with different drug concentrations, the cytoskeleton is also altered. By observing the morphological changes of cells before and after drug treatment, chemosensitivity may be assessed.

For the clinical application of the proposed method, the tumor tissue or ascites cells which purified by varies of methods 43, 44, 45 can be obtained from endometrial cancer patients. Since standard treatment for endometrial cancer patients is chemotherapy after surgery 46, 47, the tumor tissue is easy to be obtained. For patients with advanced endometrial cancer, pleural effusion and ascites are common complications and easy to be obtained in clinical routine 46. Thus, the pleural effusion and ascites can also be used to evaluate drug resistance.

## 5 CONCLUSION

In this study, the morphological parameters of endometrial cancer cells, namely, CH, CPA and CV were measured on basis of the reconstructed phase images obtained by DHM and could be used to distinguish between nondrug‐resistant ISK cells and drug‐resistant ISK‐CisR cells. Since the apoptosis activities of ISK and ISK‐CisR cells were significantly different after cisplatin treatment, the morphologies of these two cell types also exhibited related variations, including an increased height and a decreased area. Therefore, morphological changes can be used to distinguish between drug‐resistant and nondrug‐resistant cells and may be helpful for establishing new methods for detecting drug resistance of endometrial cancer cells based on biodynamic phase imaging, which may help guide chemotherapy in gynecologic oncology.

## AUTHOR BIOGRAPHIES

Please see Supporting Information online.

## Supporting information


**Appendix S1** Materials and methods.
