# Supplementary material for: An optical study of drug resistance detection in endometrial cancer cells by dynamic and quantitative phase imaging
Source: J Biophotonics. 2019 Apr 2;12(7):e201800443. doi: 10.1002/jbio.201800443 (PMC7065625; doi:10.1002/jbio.201800443)
Supplement: Supplementary file 1 — Appendix S1 Materials and methods. [file JBIO-12-e201800443-s001.docx]

| 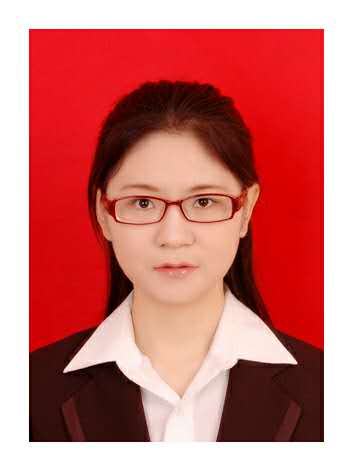 | **Mrs. Tian Yao** is currently a master student in the department of obstetrics and gynecology, Peking University People’s Hospital, Peking University, China. She received her B.S degree from South Medical University, China in 2016. Her research interest is to detect drug sensitivity of endometrial cancer. |
| --- | --- |
| 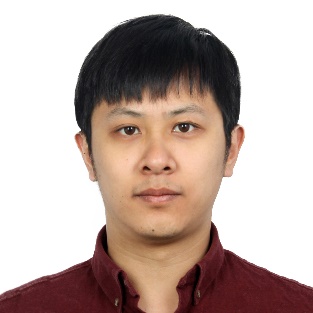 | **Mr. Runyu CAO** is a doctoral student in School of Instrumentation and Optoelectronic Engineering, Beihang University, Beijing, China. His research interests focus on applying digital holographic microscopy (DHM) to retrieve morphological and structural changing processes of living cells. He is experienced in design and optimization of optical system and digital imaging processing. |
| 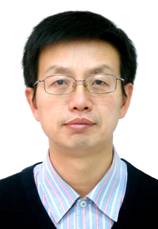 | **Prof. Wen Xiao** is the director of Key Laboratory of Optical Space, School of Instrumentation and Optoelectronic Engineering, Beihang University, Beijing, China. He received his doctoral degree of Optics Engineering in 1993 from Xi'an Institute of Optics and Precision Mechanics, Chinese Academy of Sciences. He was engaged in researches on optical fiber sensing and optical measurement for a long term. Currently, his research interests are imaging quality improvement of digital holography and the industrial and biomedical applications of digital holography. |
| 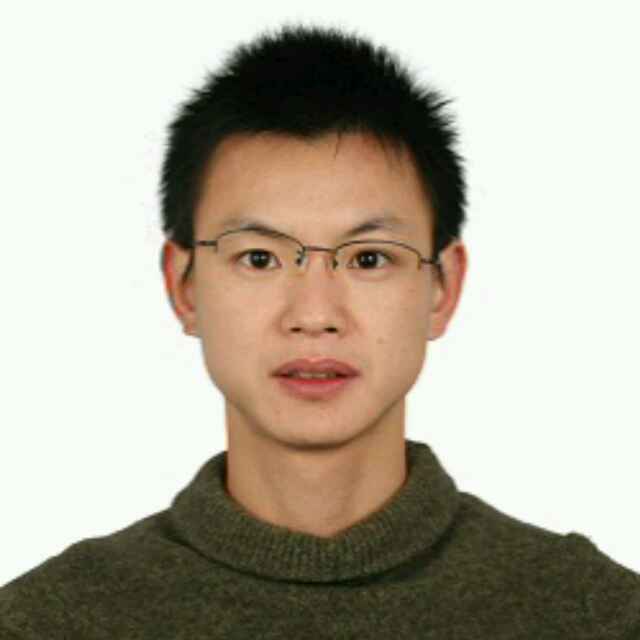 | **Dr. Feng** **Pan** is an associate professor in School of Instrumentation and Optoelectronic Engineering, Beihang University, Beijing, China. He received his doctoral degree of Physical Electronics in 2007 from Harbin Institute of Technology. His research interests are mainly about biological applications of digital holographic microscope and optical diffraction tomography, vibration measurement using holographic methods, shape measurement on large spherical surface. |
| 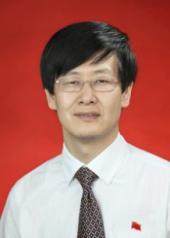 | **Xiaoping Li** received his Ph.D. from department of obstetrics and gynecology, Peking University People’s Hospital, Peking University, China. His research interest is to study the drug resistance characteristics of gynecology oncology and to approach the precise chemotherapy in gynecology oncology patients. |
